# Roles of ROS and cell cycle arrest in the genotoxicity induced by gold nanorod core/silver shell nanostructure

**DOI:** 10.1186/s11671-020-03455-1

**Published:** 2020-12-07

**Authors:** Dan Wang, Mo Dan, Yinglu Ji, Xiaochun Wu, Xue Wang, Hairuo Wen

**Affiliations:** 1 Beijing Key Laboratory, National Center for Safety Evaluation of Drugs, National Institutes for Food and Drug Control, Beijing, 100176 People’s Republic of China; 2 CAS Key Laboratory of Standardization and Measurement for Nanotechnology and CAS Center for Excellence in Nanoscience, National Center for Nanoscience and Technology, Beijing, 100190 People’s Republic of China; 3 China Pharmaceutical University, Nanjing, 211198 People’s Republic of China; 4 The State Key Laboratory of New Pharmaceutical Preparations and Excipients, 226 Huanghe Road, Shijiazhuang, 050035 Hebei People’s Republic of China; 5 National Center for Safety Evaluation of Drugs, National Institutes for Food and Drug Control, Key Laboratory of Beijing for Nonclinical Safety Evaluation Research of Drugs, Beijing, 100176 People’s Republic of China

**Keywords:** Gold nanorod core/silver shell nanostructures, Silver ions, HepaRG cells, Genotoxicity, Oxidative stress, Cell cycle arrest

## Abstract

To understand the genotoxicity induced in the liver by silver nanoparticles (AgNPs) and silver ions, an engineered gold nanorod core/silver shell nanostructure (Au@Ag NR) and humanized hepatocyte HepaRG cells were used in this study. The involvement of oxidative stress and cell cycle arrest in the DNA and chromosome damage induced by 0.4–20 µg mL^−1^ Au@Ag NR were investigated by comet assay, γ-H2AX assay and micronucleus test. Further, the distribution of Au@Ag NR was analyzed. Our results demonstrated that both Ag^+^ and Au@Ag NR led to DNA cleavage and chromosome damage (clastogenicity) in HepaRG cells and that the Au@Ag NR retained in the nucleus may further release Ag^+^, aggravating the damages, which are mainly caused by cell cycle arrest and ROS formation. The results reveal the correlation between the intracellular accumulation, Ag^+^ ion release and the potential genotoxicity of AgNPs.

## Introduction

Silver nanoparticles (AgNPs), 1 to 100 nm in size, can exhibit a broad spectrum of antimicrobial properties by penetrating pathogens and inactivating the inner sulfhydryl group of their metabolic enzymes [1]. They have demonstrated potent bacteriostasis and bactericidal effects in *Escherichia Coli*, *Neisseria gonorrhoeae* and *Chlamydia trachomatis*, and are widely employed as medical coatings, household products [2] as well as wound dressings [3]. Compelling evidence shows that nanoparticles are capable of entering the nucleus and interfering with the synthesis and transcription process of DNA [4]. In our previous study, we reported that a single intravenous dose of 5 mg/kg AgNPs could introduce remarkable chromosome breakage in the bone marrow cells of Sprague–Dawley rats [5]. A single intraperitoneal injection of 10 mg/kg or above of AgNPs induced both DNA and chromosome damage [6]. Flower et al. [7] suggested that AgNPs at doses of 50 and 100 μg mL^−1^ could trigger DNA damage within five minutes of administration, highlighting the genotoxicity of rapidly released silver (Ag). Considering the risk of excessive exposure, the investigation of NanoGenotoxicology or the DNA damage and carcinogenic potential of engineered nanomaterials has received much attention [8].

The major mechanisms for AgNP-induced genetic injuries are considered to be the overproduction of reactive oxidative species, inflammation, and cell cycle disturbance [9, 10]. As suggested in previous studies, AgNPs could either directly interact with DNA via oxidative damage [11] and interfere in the interphase at the DNA level and mitosis at the chromosomal level, or interact with the nucleoprotein and mitotic spindle apparatus to disturb cell cycle checkpoints [12]. However, whether the genotoxicity induced by AgNPs is partially attributable to the nanoparticles [13, 14] or completely to the released Ag^+^ ions is still unclear [15, 16].

Investigating the genotoxicity of AgNPs is difficult due to the unstable and uninterrupted release of the silver in the tissues, leading to difficulty in localizing the AgNPs and to differentiate the nanocore from the Ag. Our group has recently developed a gold nanorod core/silver shell (Au@Ag NR) nanostructure for studying the toxicity induced by nanoparticles [17]. The gold core of Au@Ag NR is physiologically innate in the tissue and could be used as an internal standard to monitor the release of Ag^+^ ions from the rod by monitoring the change in the Ag/Au ratio, measured using inductively coupled plasma mass spectrometry (ICP-MS) [18]. By this method, the different origins of the toxicities can be identified. Previous studies have shown that the released Ag^+^ ions from the shell of Au@Ag NR resulted in kidney oxidative damage and eventually led to morphological changes and impairment of filtration function of the glomerulus [19]. Jiang et al. [20] suggested that both particle-specific activity and intracellular silver ion release by Au@Ag NR contribute to the toxic response of granulosa cells. We also adopted Au@Ag NR as a model to study the in vivo genotoxicity potential of AgNPs and demonstrated that clastogenicity, and not mutagenicity, is the primary form of genotoxicity induced by both the Ag shell and the released Ag^+^ ions, while there was no difference in their toxicity patterns [21].

Liver is one of the major organs prone to accumulation of AgNPs and is recognized as a target organ/tissue for AgNPs-induced genotoxicity. Our previous study showed that some amount of silver (8.26 ± 3.90 μg/g) and gold (80.07 ± 64.72 μg/g) remained in the livers of SD rats eight weeks after the intravenous administration of one does of Au@Ag NR [21]. In this study, we attempted to identify the roles of cell cycle arrest and reactive oxidative stress on AgNP-induced chromosome and DNA damages using Au@Ag NR in human hepatoma-derived HepaRG cells. Genotoxicity assays, including comet assay, γ-H2AX assay and micronucleus test, were performed in parallel with oxidative radical scavenger to probe the contribution of reactive oxygen species (ROS) in DNA/chromosome damage, while the cell apoptosis, cell cycle and related proteins were determined to explore the mechanisms by which AgNPs interrupt the synthesis and replication of DNA. Further, the intracellular accumulation and distribution of Au@Ag NR was investigated by combining inductively coupled plasma mass spectrometry (ICP-MS) and transmission electron microscopy (TEM) to differentiate the role of nanoparticles and released Ag ions.

## Materials and methods

### Cell culture and treatment

Human hepatoma cell line HepaRG (Thermo Fisher Scientific) was used in this study. Cells were cultured in RPMI 1640 containing 10% fetal bovine serum (FBS, Australia Origin, Gibco) and 1% penicillin–streptomycin–glutamine solution (Gibco) in a humidified atmosphere of 5% CO_2_ at 37 °C. The cells were treated with increasing concentrations of Au@Ag NR for 24 h or 72 h, respectively, and the concentrations were determined in accordance with IC_50_ estimated by cell viability assay. To investigate the role of ROS in the genotoxicity, 1 mM *N*-Acetyl-l-cysteine (NAC, Sigma-Aldrich) was applied for 1 h prior to the treatment with Au@Ag NR.

### ATP cell growth/viability assay

The cells were seeded in a 96-well plate at a density of 5 × 10^3^/well. After 24 h of incubation, the medium was aspirated and the cells were exposed to different concentrations of Au@Ag NR for 24 h or 48 h, respectively. A broad spectrum of concentrations was prepared, and four wells per treatment were performed in one treatment period. The cytotoxicity of Au@Ag NR was examined by adenosine triphosphate (ATP) assay (CellTiter-Glo® 2.0 Assay, Promega), which measures the cellular metabolic activity by quantitating the amount of ATP, an important metabolism parameter in viable cells. The luminescent signals, which reflect the amounts of viable cells, were detected using VICTOR Multilabel Plate Reader (2030-0050, PerkinElmer), and IC_50_ values were estimated as the concentration of Au@Ag NR for half-maximal viability by Prism 7 (GraphPad Prism 7, CA, USA). The viability ratio is calculated using the following equation:

$${\text{Viability}}\,{\text{Ratio}}\,\left( \% \right) = {\text{RLU}}_{{{\text{sample}}}} /{\text{RLU}}_{{{\text{vehicle}}}} \times {1}00\%$$

where RLU is the relative light unit represented as the mean value of four wells, RLU_vehicle_ represented cells not treated with nanorods, and RLU_sample_ represented cells that were treated with different concentrations of Au@Ag NR.

### Concentration determination of silver and gold in cells

The cell samples were digested in nitric acid using the microwave digestion system. Following the digestion, the samples were prepared with a mixture containing 1% nitric acid and hydrochloric acid. The quantities of Ag and Au in the solutions were determined by ICP-MS (NexION300X, PerkinElmer). TEM analysis was used to determine the presence of Au NR and Au@Ag NR in the cell. The cell samples were fixed in a mixture of 2.5% glutaraldehyde and 2% paraformaldehyde for 2 h at 4 °C. The cell pellets were fixed and rinsed three times in phosphate buffer (pH 7.4) and post-fixed in 1% osmium tetroxide for 2 h at 4 °C. The samples were subsequently rinsed in distilled water three times and dehydrated for 15 min in different concentrations of ethanol (50%, 70%, 90% and 100% ethanol, respectively) one after the other. Subsequently, propylene oxide at 1:1 and 1:3 dilutions was applied to the resin at 20–26 °C for 2 h. Polymerization was performed by graded heating at 35 °C for 16 h, 45 °C for 8 h, 55 °C for 14 h and 65 °C for 48 h. Ultrathin sections were stained for 25 min with uranyl acetate and lead citrate and analyzed by a transmission electron microscope (H-7650, HITACHI, Japan).

### Conventional and modified comet assay

The cells were seeded in 12-well plates at densities of 2 × 10^5^/well or 3 × 10^5^/well for a 24- or 72-h treatment, respectively. Hydrogen peroxide (H_2_O_2_) at a concentration of 200 μmol was exposed to the cells as positive control for an hour. For each sample, two wells were prepared for both the conventional treatment and the formamidopyrimidine glycosylase (Fpg) treatment. Conventional comet assay was performed in alkaline conditions (pH > 13) as described previously [21]. For the Fpg-treated wells, an additional Fpg treatment was applied before the DNA unwinding procedure, and the slides were immersed in an enzyme buffer (0.1 M KCl, 0.5 mM EDTA, 40 mM HEPES, 0.2 mg.mL^−1^ BSA) three times for 5 min each. The Fpg (New England Biolabs, Inc., UK) was diluted at 1:50,000 with enzyme buffer. One hundred milliliter aliquots of the diluted enzyme were added to each gel on the microscope slides and incubated in a humidity chamber at 37 °C for 30 min. The remaining steps were the same as the conventional treatment. The comet assays were performed in triplicate. At least 50 cells per sample were independently scored using the Nikon Eclipse 80i fluorescent microscope (Nikon, Tokyo, Japan), while Komet 6.0 (Andor Technology, Belfast, UK) was used to analyze the medium value of percentage DNA in tail and olive tail moment (OTM) of each sample.

### Qualification of γ-H2AX foci by flow cytometry and high-content screening

For the quantification using flow cytometry, cells were seeded in 12-well plates at densities of 2 × 10^5^/well or 3 × 10^5^/well for a 24- or 72-h treatment, respectively, while for the high-content screening assay, cells were seeded in 96-well plates at densities of 6 × 10^3^/well or 1 × 10^4^/well for a 24- or 72-h treatment, respectively. As a positive control, 2 μM methyl methanesulfonate (MMS, Sigma-Aldrich) was applied in parallel with the cells for an hour. The cells were rinsed in tris-buffered saline (TBS) and fixed with 4% paraformaldehyde for 15 min at room temperature. After washing with TBS, the cells were incubated with 50 μL ice-cold methanol for 30 min at − 20 °C. The cells were further rinsed in TBS three times, and the blocking reagent (TBS containing 0.3% Triton X-100 and 10% goat serum) was applied for 1 h. The primary antibody (mouse anti-phospho-H2AX Ser139, Millipore) was diluted to 1:200 with blocking reagent and incubated with the cells overnight at 4 °C. The plate was then again rinsed with TBS for three times, and the secondary antibody (Alexa Fluor 488 goat anti-mouse, Life Technologies), diluted with the blocking reagent in 1:20 ratio, was added subsequently. The samples was kept in the dark at room temperature for 1 h, and 2 μg mL^−1^ (20 μL/well) DAPI (Invitrogen) was added to each well. The fluorescence was measured using a flow cytometry (FACSCalibur, BD Bioscience, NJ, USA) or High Content Analysis System (Operetta CLS, PerkinElmer). For the flow cytometry assay, data from at least 10,000 cells per group were analyzed, and the experiments were performed in triplicate; for high-content analysis, 20 visual fields in each well and at least five wells in each group were analyzed.

### Cytokinesis-block micronucleus cytome (CBMN-cyt) assay

CBMN-cyt was performed according to the procedure described by Fenech et al. [22]. Cells were seeded in 12-well plates at densities of 2 × 10^5^/well or 3 × 10^5^/well for a 24- or 72-h treatment, respectively. 0.2 μg mL^−1^ Mitomycin C (MMC, Tokyo Chemical Industry Co., Ltd. Japan) was exposed to the cells as positive control for 24 h. 3 μg mL^−1^ cytochalasin B was applied after a 24- or 72-h treatment to block the cytokinesis process, and the cells were harvested after 40 h. The samples were stained with 5% Giemsa after hypotonicity with pre-warmed 0.075 mol L^−1^ KCl and fixation with a 3:1 mixture of methanol and acetic acid. Triplicate wells per group were prepared, and at least 1000 binucleate cells per well were examined.

### Measurement of MDA, total GSH and SOD contents

The cells were cultured in 12-well plates at densities of 5 × 10^5^/well or 3 × 10^5^/well for a 24- or 72-h treatment, respectively. Subsequently, the cells were harvested and rinsed three times with phosphate buffer saline (PBS). The amounts of malondialdehyde (MDA) in the cell homogenates were determined using a thiobarbituric acid-based method (Nanjing Jiancheng Bio-engineering Institute, Nanjing, China). The amounts of total glutathione (GSH) and superoxide dismutase (SOD) were determined using the total glutathione quantification and SOD assay kits (Dojindo Molecular Technologies, Inc. Kumamoto, Japan), respectively. Optical densities (O.D) of each well was measured using VICTOR Multilabel Plate Reader (2030-0050, PerkinElmer).

### Flow cytometric analysis for cell cycle

The cells were cultured in 6-well plates at densities of 1 × 10^6^/well or 5 × 10^5^/well for a 24- or 72-h treatment, respectively, and were subsequently fixed with 70% ethanol at 4 °C overnight. The samples were rinsed with PBS three times and stained with PI/Rnase staining buffer (BD Biosciences) for 15 min at room temperature. Cell populations under G0/G1, S and G2/M phase among 20,000 cells were determined by employing regions with FL2 area versus FL2 width. Analysis was done by flow cytometry (FACSCalibur, BD Bioscience, NJ, USA) and FlowJo (BD Bioscience), and the experiments were performed in triplicate.

### Flow cytometric analysis of cell apoptosis

The cells were cultured in 6-well plates at densities of 1 × 10^6^/well or 5 × 10^5^/well for a 24- or 72-h treatment, respectively. They were subsequently rinsed twice with PBS and diluted with 500 μL 1 × binding buffer (FITC Annexin V Apoptosis Detection Kit I, BD Bioscience) to adjust the suspension to around 1 × 10^6^ cells/mL, and subsequently 100 μL dilution was mixed with 5 µL FITC Annexin V and 5 µL PI. The samples were stained at room temperature for 15 min, and at least 10,000 cells were analyzed to determine the cell population under early and late apoptosis by employing regions with FL1H versus FL2H using flow cytometry (FACSCalibur, BD Bioscience, NJ, USA) and FlowJo (BD Bioscience). The experiments were performed in triplicate.

#### Western blot analysis

The cells were cultured in a 75-cm^2^ flask at densities of 1 × 10^7^/well and 6 × 10^6^/well for a 24- and 72-h treatment, respectively. The cells were lysed with RIPA lysis buffer containing protease inhibitor (PMSF), and the concentration of proteins was determined using a BCA protein quantification kit (Beyotime Biotechnology, China). The concentrations of the samples were adjusted using RIPA lysis buffer prior to denaturation by heating at 95 °C for 3 min. The protein samples were separated by electrophoresis on 12% SDS polyacrylamide gels and transferred to nitrocellulose membranes (Millipore). The membranes were blocked with 5% skim milk for 30 min and incubated with primary p53 (SC-137174,Santa Cruz), p21 (SC-6246, Santa Cruz) and β-actin (sc-47778, Santa Cruz) and secondary antibodies goat anti-mouse IgG(H+L)-HRP(SE131, solabio), respectively. The expression levels of the target proteins in the samples were visualized using an enhanced chemiluminescence (ECL) method and analyzed by ImageJ system (National Institutes of Health).

#### Statistical analyses

The data were presented as the mean ± SEM. One-way analysis of variance (ANOVA) was used to test statistical significance of differences among negative control and treated groups, followed by the Dunnett multiple comparison test using SPSS (version 22, IBM, Armonk, NY, USA), and data were considered statistically significant at *P* < 0.05. The figures were prepared using GraphPad Prism 7 for Windows (GraphPad Software, La Jolla, CA, USA).

## Results

### Characterization of Au NR and Au@Ag NR

**Fig. 1:**
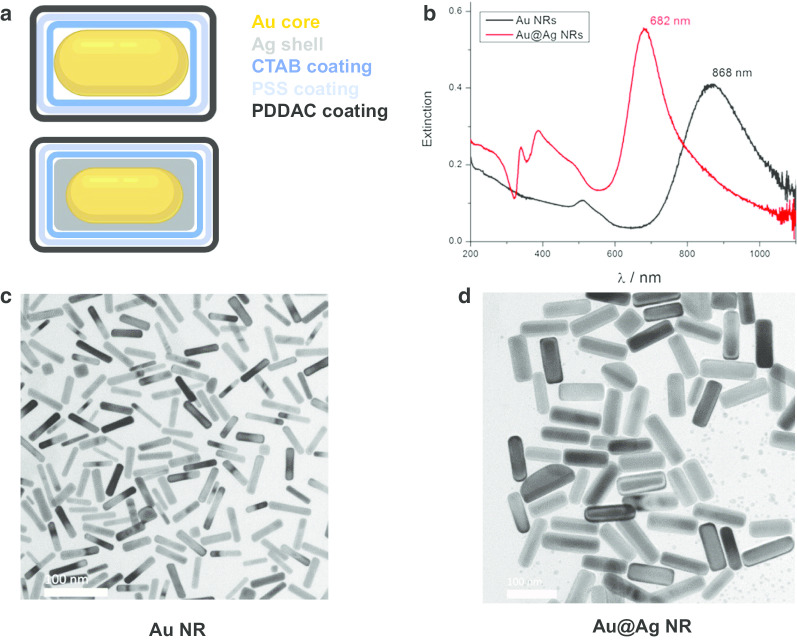
Characterization of Au NR and Au@Ag NR. **a** Structural diagram of Au NR and Au@Ag NR; **b** UV–Vis–NIR extinction spectra of Au NR and Au@Ag NR dispersed in water; **c** representative TEM images of Au NR; **d** representative TEM images of Au @Ag NR

Gold nanorods (Au NRs), gold nanorod cores and silver shell nanostructures (Au@Ag NR) were engineered, prepared and characterized as previously described [21]. Briefly, the mean diameters and lengths are 15.0 ± 2.5 nm, 66.7 ± 2.5 nm for Au NRs and 26.2 ± 3.0 nm, 72.7 ± 8.9 nm for Au@Ag NRs. The Ag shell thickness is about 5 nm. The zeta potentials of PDDAC-coated Au NRs and Au@Ag NRs dispersed in water were 37.7 ± 1.6 mV and 52.5 ± 1.4 mV, respectively. The Ag/Au weight ratio of prepared Au@Ag NR was estimated as 2.3. The characterization results are shown in Fig. 1.

### Cell viability

The cytotoxicity of Au@Ag NR toward HepaRG cells was investigated by ATP viability assay (Table 1), and the cells were exposed to Au@Ag NR for 24 or 48 h at concentrations varying from 0.125 to 160 μg mL^−1^. Au@Ag NR induced significant cytotoxic effects in both time-and dose-dependent manners after exposure of 24 and 48 h, with % viability IC_50_ at 20 µg mL^−1^ and 6 µg mL^−1^, fitted by the software GraphPad Prism 7.0, respectively. Considering the overall cytotoxicity, the treatment periods were adjusted to 24 h and 72 h, while the concentrations applied were determined to be 0.8 µg mL^−1^, 4 µg mL^−1^ and 20 µg mL^−1^. In addition, Au NR was included as an inert control, and the Au content in the AuNR group was the same as 20 µg mL^−1^ Au@Ag NR, which is 16 µg mL^−1^. In contrast, 1 mM NAC pretreatment was adopted in the Au@Ag NR + NAC group as a control for oxidative stress response (the concentration of Au@Ag NR is 20 µg mL^−1^).

**Table 1:** Cytotoxic potential of Au@Ag NRs in HepaRG cells after 24 and 48 h of exposure

	Concentrations (μg mL^−1^)								
	160	80	40	20	10	5	2.5	1.25	0.125
24 h %viability	0.52 ± 0.19	0.66 ± 0.18	45.47 ± 1.31	68.72 ± 1.74	74.62 ± 0.65	76.42 ± 2.20	80.86 ± 1.63	83.44 ± 0.77	85.12 ± 1.59
48 h %viability	0.22 ± 0.05	1.08 ± 0.52	22.39 ± 1.41	50.27 ± 1.88	60.44 ± 2.02	65.41 ± 1.45	68.42 ± 1.25	74.86 ± 0.60	96.09 ± 0.12

Mean ± SEM, *n* = 4

### Cell distribution of Au NR and Au@Ag NR

**Fig. 2:**
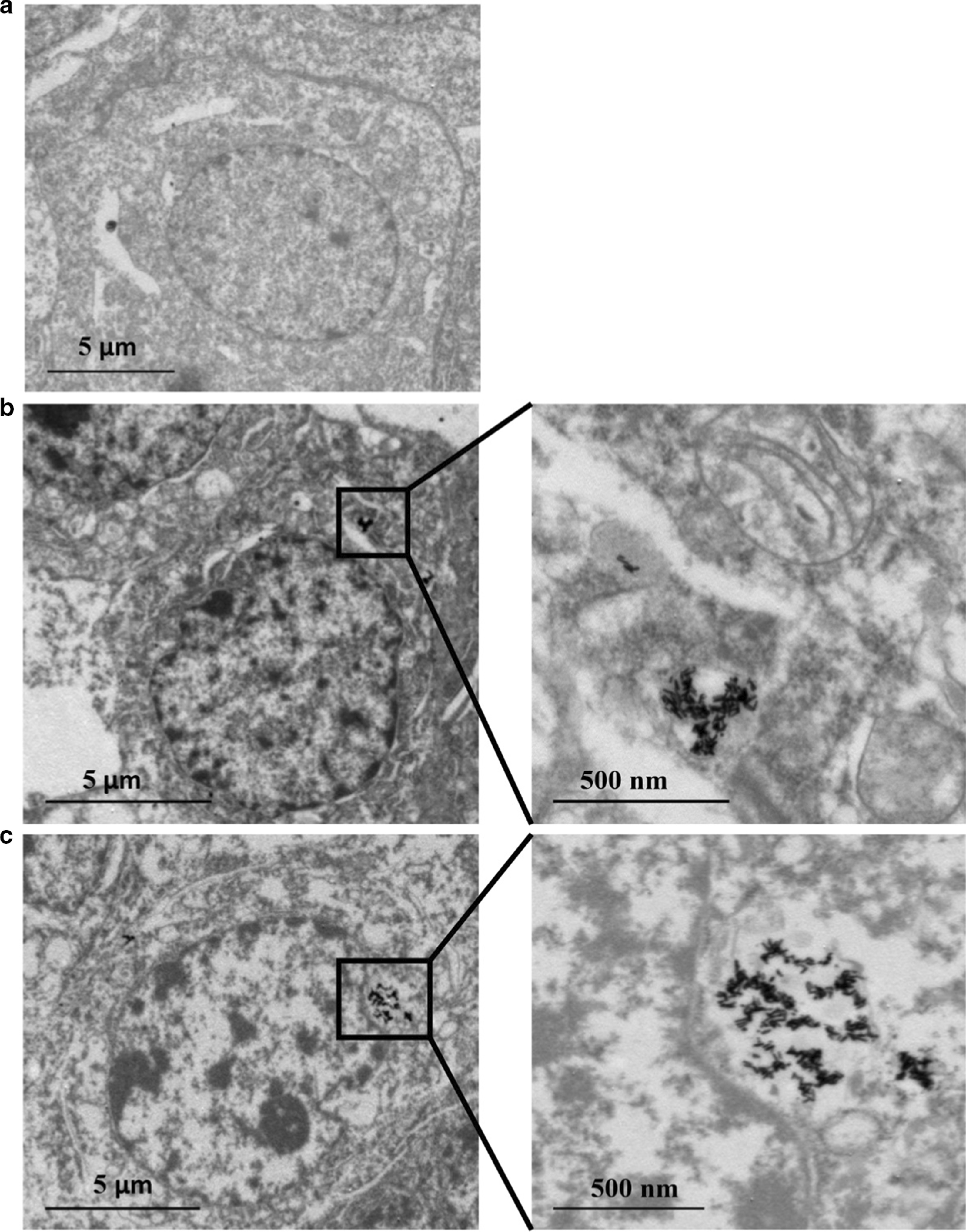
Au NR and Au@Ag NR internalization: HepaRG by TEM at 80 kV after 24 h of exposure to 16 μg mL^−1^ Au NR and 20 μg mL^−1^ Au@Ag NR. **a** Vehicle control; **b** Au NR; **c** Au@Ag NR

The distribution of Au and Ag content in the HepaRG cells was analyzed by ICP-MS. As shown in Tables 2 and 3, the Ag content increased in a dose-dependent manner. However, the antioxidant *N*-Acetyl-l-cysteine (NAC) as free radical scavenger may restrict the cellular uptake of nanoparticles, as lesser Ag content was observed even though the same concentration of Au@Ag NR (20 µg mL^−1^) was applied in this group. The decline in Ag/Au ratio from 24 to 72 h indicated a continuous release of Ag^+^ from the shell of Au@Ag NR. Also, the cellular uptake of Ag is much more than Au (Table 4). Furthermore, TEM data showed that most of the Au NR and Au@Ag NR were retained in the cells as agglomerates. The structures of nanorods were clearly seen inside the cells subject to the exposure of Au NR or Au@Ag NR without entering the nucleus (Fig. 2).

**Table 2:** Intracellular levels of Au and Ag

	Concentration of Ag (μg/mg protein)		Concentration of Au (μg/mg protein)	
	24 h	72 h	24 h	72 h
Au 16 μg mL^−1^	–	–	1.46	7.47
Au @Ag NRs 0.8 μg mL^−1^	1.63	2.14	0.19	0.38
Au @Ag NRs 4 μg mL^−1^	5.34	8.08	0.59	3.49
Au @Ag NRs 20 μg mL^−1^	27.56	54.12	14.40	33.91
Au @Ag NRs + NAC	19.33	30.46	10.07	32.48

**Table 3:** Weight ratio of Ag/Au

	Ag/Au	
	24 h	72 h
Au @Ag NRs 0.8 μg mL^−1^	15.6	10.3
Au @Ag NRs 4 μg mL^−1^	16.5	4.2
Au @Ag NRs 20 μg mL^−1^	3.5	2.9
Au @Ag NRs + NAC	3.5	1.7

**Table 4:** % Cell uptake of Ag and Au

	Ag		Au	
	24 h	72 h	24 h	72 h
Au 16 μg mL^−1^	–	–	2.49	8.77
Au @Ag NRs 0.8 μg mL^−1^	48.79	41.32	7.38	9.35
Au @Ag NRs 4 μg mL^−1^	40.11	30.24	5.54	16.35
Au @Ag NRs 20 μg mL^−1^	31.57	30.26	20.62	23.71
Au @Ag NRs + NAC	31.3	16.86	20.37	22.48

### DNA damage

**Fig. 3:**
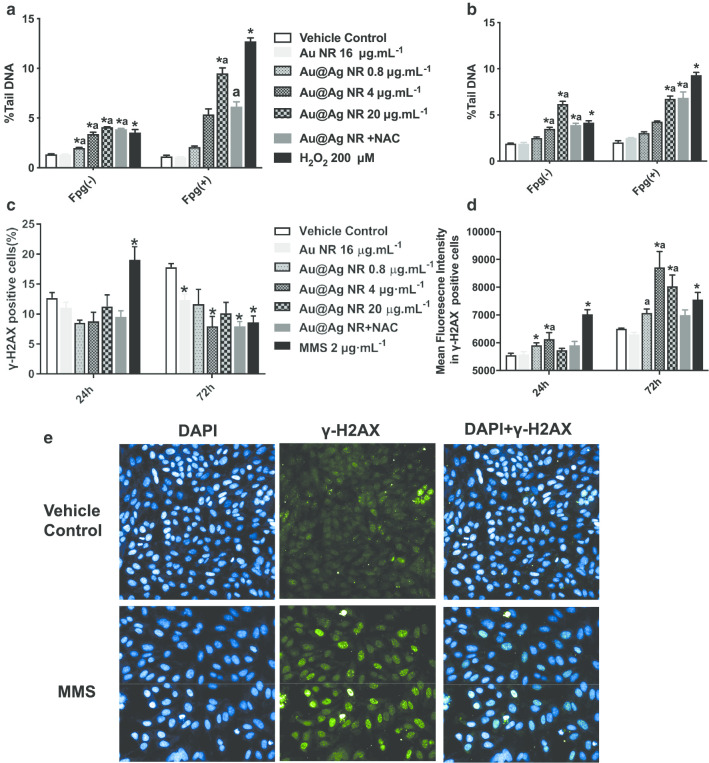
DNA damage induced by Au@Ag NR. HepaGR cells were exposed to Au@Ag NR at different concentrations (0.8 to 20 μg mL^−1^) for 24 h and 72 h, respectively. **a** Averaged % Tail DNA after exposed to Au@Ag NR for 24 h; **b** averaged % Tail DNA after exposed to Au@Ag NR for 72 h; **c** percentage of positive cells with γ-H2AX foci estimated using flow cytometry; **d** mean fluorescence intensities in cells with γ-H2AX foci estimated using immunofluorescent staining.^*^P < 0.05 versus vehicle control; ^a^*P* < 0.05 versus Au NR. 2 μM mL^−1^ MMS was employed as a positive control

The DNA damage triggered by Au@Ag NR was evaluated by both comet assay and γH2AX assay (Fig. 3). It was observed from comet assay that 0.8 to 20 µg mL^−1^ Au@Ag NR could introduce significant DNA damage. After a 24- or 72-h exposure to Au@Ag NR, both % tail DNA and OTM of cells increased in both time- and concentration-dependent manners. In addition, DNA damage associated with oxidative stress induction was observed in the cells treated with 20 µg mL^−1^ Au@Ag NR by the Fgp enzyme-modified comet assay (Fig. 3a, b). For evaluating the extent of double-strand breakage which represents a higher correlation to the genesis of cancer, both γ-H2AX-positive cells and mean fluorescence intensities in γ-H2AX-positive cells were analyzed. After a 24-h exposure to Au@Ag NR, no difference was found among groups in γ-H2AX-positive cells. However, 4 μg mL^−1^ Au@Ag NR group caused a significant increase after a 72-h treatment. Significant increases in fluorescence intensities were observed in all Au@Ag NR groups after 72 h compared with the vehicle control (Fig. 3c–e, *P* < 0.05).

### Chromosomal damage

**Fig. 4:**
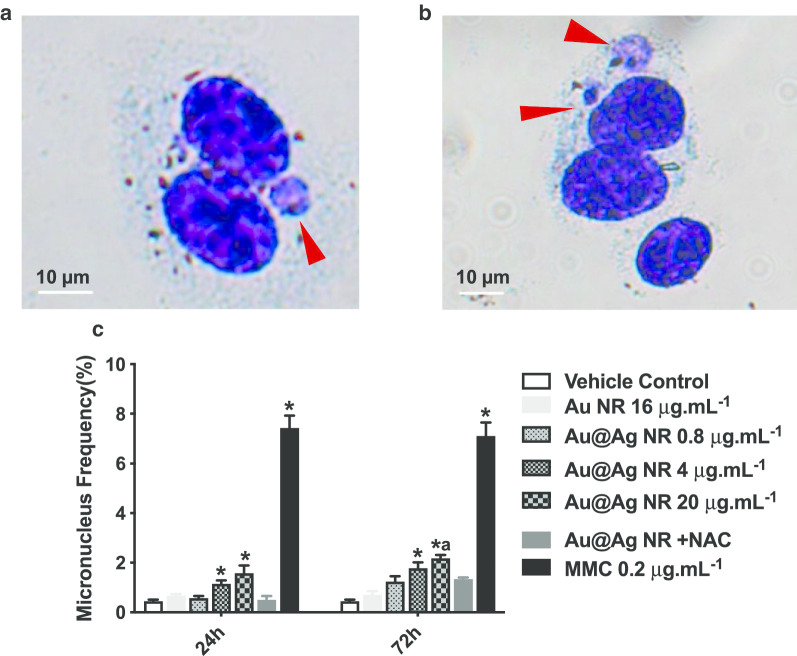
Chromosome damage induced by Au@Ag NR. HepaGR cells were exposed to Au@Ag NR at different concentrations from 0.8 μg mL^−1^ to 20 μg mL^−1^ for 24 h and 72 h. **a**, **b** Representative images of micronucleus (red arrow); **c** micronucleus frequency (%). ^*^P < 0.05 versus vehicle control; ^a^*P* < 0.05 versus Au NR. 0.2 μg mL^−1^ mitomycin C was employed as a positive control

The formation of micronuclei is a significant biomarker for identifying chromosomal damage, which is a more critical damage to the genetic material than DNA breakage. The ratio of binucleated cells containing micronucleus was scored as shown in Fig. 4c. Au@Ag NR increased the micronucleus formation in a concentration-dependent pattern. After a 24-h exposure, the ratios of micronucleus observed in cells treated with 4 μg mL^−1^ Au@Ag NR and 20 μg mL^−1^ Au@Ag NR were 1.133 ± 0.145% and 1.567 ± 0.318%, respectively, both of which were significantly higher than those in the vehicle control group. After a 72-h exposure, the ratio of micronucleus in cells treated with 4 μg mL^−1^ Au@Ag NR was 1.767 ± 0.233%, which was significantly higher than the vehicle control group; the ratio of micronucleus in cells treated with 20 μg mL^−1^ Au@Ag NR was 2.167 ± 0.252%, which was significantly higher than those observed in both vehicle control group and 16 μg mL^−1^ Au NR group (0.700 ± 0.153%). In contrast, no difference was found between cells treated with 20 μg mL^−1^ Au@Ag NR + NAC and vehicle control, suggesting the participation of ROS in the chromosome breakage induced by Au@Ag NR.

### Effects of Au@Ag NR on the ROS Formation

**Fig. 5:**
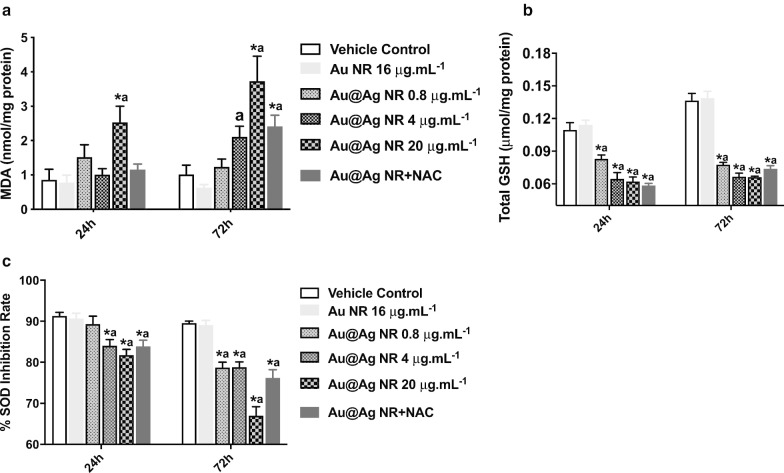
Effects of Au@Ag NR on the ROS formation. HepaGR cells were exposed to Au@Ag NR at different concentrations from 0.8 μg mL^−1^ to 20 μg mL^−1^ for 24 h and 72 h. **a** MDA level; **b** GSH level; **c** SOD level. ^*^P < 0.05 versus vehicle control; ^a^*P* < 0.05 versus Au NR

To further explore the role of ROS formation in Au@Ag NR-induced DNA and chromosome damages, MDA, GSH and SOD levels were estimated. A significant increase in MDA formation (*P* < 0.05) was observed after exposure to 20 μg mL^−1^ Au@Ag NR for both 24 and 72 h (Fig. 5a). Further, the GSH and SOD levels in cells exposed to Au@Ag NR showed significant reduction (*P* < 0.05) in a time- and concentration-dependent manner. These results suggested an imbalance between oxidation and anti-oxidation, generated by the exposure of Au@Ag NR (Fig. 5b, c).

### Effects of Au@Ag NR on the cell cycle and apoptosis

**Fig. 6:**
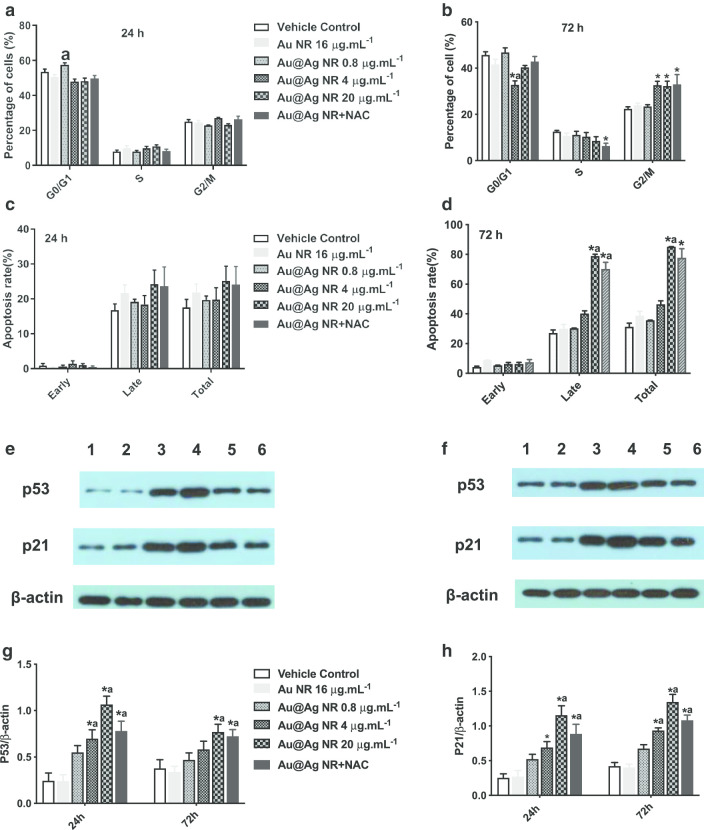
Effects of Au@Ag NR on the cell cycle and apoptosis. Effects of Au@Ag NR on cell cycle (**a**, **b**) and apoptosis (**c**, **d**) after exposed for 24 h and 72 h, respectively; the representative data of expression levels of p53 and p21 in HepaRG cells of different groups (**e**, **f** Lane 1: vehicle control; Lane 2: Au NR; Lane 3: Au@Ag NR + NAC; Lane 4: Au@Ag NR 20 μg mL^−1^; Lane 5: Au@Ag NR 4 μg mL^−1^; Lane 6:Au@Ag NR 0.8 μg mL^−1^); the averaged relative expression level of p53 and p21 to β-actin in different groups was summarized in (**g**, **f**).^*^P < 0.05 versus vehicle control; ^a^*P* < 0.05 versus Au NR

After a 72-h exposure to Au@Ag NR, the increase in numbers of cells in phase G2/M was observed in 4 μg mL^−1^ Au@Ag NR, 20 μg mL^−1^ Au@Ag NR and Au@Ag NR + NAC group, with proportions of 32.63% ± 1.77%, 32.267% ± 2.17% and 32.967% ± 4.25%, respectively (Fig. 6a, b), which were significantly greater than those in the vehicle control group (22.37% ± 0.92%). In the meanwhile, cell apoptosis induced by Au@Ag NR could be observed after a 72-h exposure, and the late apoptosis rate of cells treated with 20 μg mL^−1^ Au@Ag NR and 20 μg mL^−1^ Au@Ag NR + NAC was 78.90 ± 1.19% and 70.20 ± 4.50%, respectively (Fig. 6c, d). Au@Ag NR induced more late apoptosis than early apoptosis, and the treatment of NAC could alleviate the cell rate of late apoptosis triggered by Au@Ag NR.

The expression levels of p21 and p53 were detected by Western blots, and a similar pattern was observed. The expression levels of p53 and p21 in cells treated with 4 μg mL^−1^ and 20 μg mL^−1^ Au@Ag NR were markedly increased (*P* < 0.05) and were significantly decreased in cells treated with both 20 μg mL^−1^ Au@Ag NR and NAC (*P* < 0.05, compared with 20 μg mL^−1^ Au@Ag NR group, Fig. 6e–h). It is known that p53 protein is a core molecule mediating G2/M checkpoint activation in response to DNA damage, and p21 is recognized as a p53-dependent cell cycle inhibitor. Thus, the Au@Ag NR could interfere with DNA replication and hinder the DNA repair by the cell cycle arrest.

## Discussion

At present, the roles of the released Ag^+^ and AgNPs in generating genotoxicity are far from clear. Previous studies from our group [21] and others [13] have demonstrated that while Ag^+^ is the major source for introducing toxicities, nanoparticles could also be highly toxic. For instance, AgNPs could contribute to the genotoxicity by inducing the formation of hydroxyl radicals [13]. Further, more severe chromosome damage, oxidative stress and apoptosis were introduced by AgNP compared to Ag^+^ alone [23], suggesting that different pathways might be involved. We employed Au@Ag NR as a model material to understand the forms and distributions of AgNPs in cells, and the amounts of intracellular Ag and Au were determined by ICP-MS. The Ag/Au weight ratio of prepared Au@Ag NR was estimated as 2.3. However, after a 24-h exposure, it sharply increased to 16.5 in the cells treated with Au@Ag NR, suggesting that large amount of Ag was released from the shell of Au@Ag NR within that period. When the exposure period of Au@Ag NR was extended to 72 h, the Au/Ag weight ratio was decreased to 1.7, indicating that the Ag^+^ was released from the cell and the nanorod was the major form of Au@Ag NR in the cell at that stage. Therefore, it could be deduced that once the Au@Ag NR entered the cell, Ag^+^ rapidly dissolved from its shell within 24 h and gradually released to the extracellular environment, while the Au@Ag NR itself retained in the cell for a longer period.

Oxidative stress is deemed as one of the most important toxicological mechanisms of nanoparticles [24]. N-acetylcysteine (NAC) is a thiol, a mucolytic agent and precursor of l-cysteine which reduced glutathione. NAC is also a source of sulfhydryl groups in cells and exerts the ROS scavenger activity by interacting with OH^**·**^ and H_2_O_2_[25]. In this study, the GSH and SOD levels were significantly decreased after exposure to Au@Ag NR, while the MDA level increased in a concentration- and time-dependent manner, indicating that the Au@Ag NR introduced the oxidative stress in the cells.

The potentials of Ag and Au@Ag NR in interfering with the genetic materials were further investigated by a series of genotoxicity assays. It is noteworthy that co-culturing the NAC with Au@Ag NR could ameliorate the ROS formation, which in turn supports the participation of oxidative stress in the genotoxicity triggered by Au@Ag NR. In this study, comet and γ-H2AX assays were performed to confirm that Au@Ag NR could interact with DNA and induce certain DNA damage, and the repair endonuclease Fpg was included in the comet assay to identify the oxidative DNA damage [26]. The Fgp could recognize oxidized pyrimidines and remove oxidized purines, e.g., 8-hydroguanine, so as to create apurinic or apyrimidinic sites that could introduce gaps in the DNA strands. The oxidative stress-induced DNA breakage could be determined subsequently by another comet assay [27]. The further DNA breakage detected by the additional Fgp in the comet assay suggested that the Au@Ag NR could cause DNA damage. Mei et al. [28] observed that 5-nm-sized AgNPs induced oxidative lesion-specific DNA damage by employing the hOGG1, EndoIII and Fpg endonucleases in the comet assay. Li et al. [29] also suggested that both PVP- and silica-coated AgNPs (15–100 nm and 10–80 nm, respectively) could lead to a significant increase in DNA breakage in mice hepatocytes in the presence of hOGG1and EndoIII. The formation of γ-H2AX foci, which represents an early cellular response to genotoxic stress, is the most sensitive and specific biomarker for detecting DSBs [30]. As demonstrated in this study, γ-H2AX foci in cells exposed to Au@Ag NR were markedly increased after 24 h, and a further increase could be observed after 72 h. The reduction in the 20 µg mL^−1^ group might be due to the cytotoxicity to the HepaRG cells at higher concentration. Similar results were observed for AgNPs with different coatings [31, 32]. Further, our results suggest that Au@Ag NR could induce chromosome damage in HepaRG cells, as the micronucleus rates were significantly increased. This is consistent with previous studies, where AgNPs-induced increased micronucleus rate was reported in HaCaT and TK6 cells [33]. In contrast, the addition of oxidative radical scavenger NAC could inhibit the formation of micronucleus induced by Au@Ag NR. Taken together, these data suggest the participation of oxidative stress in AgNP-introduced clastogenicity risk in vitro.

Previous studies have investigated the cell cycle arrest and cytotoxicity induced by AgNPs [33–35]. With prolonging the exposure time, the impact of AgNPs on cell cycle and apoptosis might be enhanced and in turn aggravate the cytotoxicity and genotoxicity. Usually, the cell cycle checkpoints (e.g., G2/M) were initiated by cells when experiencing DNA damage, and this mechanism serves to prevent the cell from entering mitosis (M phase). The G2/M cell cycle arrest indicates that an increasing percentage of cells is hindered in G2 phase for DNA repairing. Cells experiencing successful DNA repairing would further proceed to mitosis; however, for those with fatal damages, irreversible G2/M cell cycle arrest and cells apoptosis would take place [36]. We observed that Au@Ag NR could arrest the majority of HepaRG cells in G2/M phase, induce late cell apoptosis and increase the expression levels of p53 and p21, which are important proteins associated with the regulation of cell cycles [37]. As p53 could also induce apoptosis, when the DNA cannot be repaired properly [38], the p21 might indirectly participate in cell apoptosis by cell cycle arrest in a p53-dependent pathway via down-regulating the nuclear protein ICBP90 for DNA replication and cell cycle regulation [39]. Furthermore, apoptosis and a G2/M arrest induced by activation of the p53/p21 system have been reported in HepG2 cells following the administration of garlic extracts [40]. Thus, it could be inferred that the oxidative stress-triggered DNA/chromosome damages might facilitate the expression of p53 and p21, which subsequently induces cell cycle arrest. Extending the exposure period of Au@Ag NRs to the DNA/chromosome during replication may further aggravate the genotoxicity or apoptosis.

## Conclusion

**Fig. 7:**
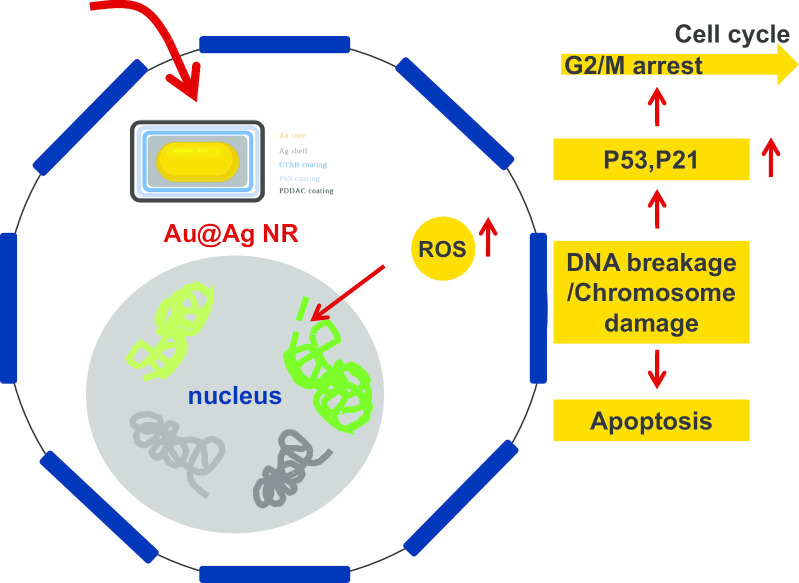
Schematic diagram of the possible mechanism of genotoxicity introduced by AgNP in vitro

Genotoxicity induced by AgNPs may be attributed to the oxidative stress induced by the nanoparticles as well as the released ions [41]. This study employed Au@Ag NR as a model to determine the distribution and release behavior of Ag after the nanoparticles enter into the cells. Considering the disparate forms of Au@Ag NR in the cell, after its exposure the Ag^+^ was rapidly dissolved from the silver shell. Ag^+^ and Au@Ag NR could introduce cytotoxicity and genotoxicity (clastogenicity) in the cells, and the Au@Ag NR retained in the nucleus may further release Ag^+^ to aggravate the damage, which are mainly caused by cell cycle arrest and ROS formation (summarized in Fig. 7). Collectively, these data reveal the correlation between the intracellular accumulation, Ag^+^ release as well as the potential genotoxicity of AgNPs.

## Data Availability

All data and materials are available without restriction.
